# Preparation and Improved Properties of Vanillin-Crosslinked Polyvinyl Alcohol/Chitosan Active Packaging Films

**DOI:** 10.3390/molecules30061334

**Published:** 2025-03-16

**Authors:** Yina He, Xiaojun Zhang, Zhan Zhang, Bing Lin, Haitao Yu

**Affiliations:** 1 Zhejiang Marine Fisheries Research Institute, Zhoushan 316021, China; 2 Key Laboratory of Sustainable Utilization of Technology Research for Fisheries Resources of Zhejiang Province, Zhoushan 316021, China; 3 Food and Pharmacy College, Zhejiang Ocean University, Zhoushan 316022, China; 4 Zhejiang Retronx Foodstuff Industry Co., Ltd., Zhoushan 316104, China

**Keywords:** vanillin, polyvinyl alcohol, chitosan, active packaging, composite film

## Abstract

Using chitosan and polyvinyl alcohol (PVA) as substrates, active packaging composite (VPC) films based on vanillin-crosslinked PVA/chitosan with different concentrations of added vanillin were prepared and characterized. The results show that the VPC films exhibited higher tightness and hydrophobicity, lower water content and water vapor permeability, as well as better UV resistance. The potential interactions between the film groups were analyzed by SEM, FTIR, and XRD, and the results showed that the formation of Schiff base and hydrogen bond interactions affected the properties of the films. The VPC films also showed excellent antioxidant activity. Therefore, vanillin-crosslinked PVA/chitosan active films have broader application prospects as packaging materials for food preservation.

## 1. Introduction

As the economy and society progress, food safety issues have received increasing attention, and the focus of the human diet has shifted from quantity to quality, toward healthier and safer food products. The emphasis on food safety has changed the requirements for food packaging, focusing on effective packaging materials; this has promoted the development of the food packaging industry, with increasing attention to the safety of the materials involved [1]. Food packaging film is ubiquitous in daily life; its main function is to wrap the surface of the food product, separating it from external bacteria and pollutants to prevent its deterioration [2]. Most of the traditional food packaging films are made of petroleum-based plastic materials, whose widespread use reflects their excellent packaging properties, mature production processes, and low manufacturing costs. However, these packaging materials are difficult to degrade in the natural environment, and the related waste will lead to environmental pollution and even precipitation of toxic substances, thus affecting the ecological balance [3]. Therefore, biodegradable materials have become a research hotspot in the field of food packaging.

Biodegradable materials have several advantages, such as improved food safety, preserved food quality, and extended shelf-life, and at the same time address environmental issues associated with non-biodegradable plastics [4]. Studies have found that biodegradable packaging films suffer from high production costs along with poor mechanical and barrier properties and are subject to certain restrictions in the food packaging industry [3]. In order to eliminate the bottleneck of film production, biobased films are usually modified to improve their performance and meet different packaging requirements. Therefore, the development of efficient, biodegradable, and non-toxic materials for food packaging films has attracted increasing research attention.

Chitosan (CS) is a hydrophobic cationic polysaccharide composed of *N*-acetyl-D-glucosamine and D-glucosamine units. It can be prepared by partial deacetylation of chitin [5] and is one of the most abundant polysaccharides in nature [6]. This unique biopolymer is commercially produced from shellfish-processing waste, with advantageous features including a low price, easy availability, a wide range of sources, safety, and biodegradability [7]; moreover, it contains hydroxyl groups and has good thermal stability [8]. However, pure chitosan films suffer from problems such as poor mechanical properties along with low water and oxidation resistances, which prevent them from meeting the requirements for practical applications. Polyvinyl alcohol (PVA) is a water-soluble and non-toxic polymer [9] with good mechanical, processability, bonding, film-forming, and biodegradability properties, which can be blended with natural polymer materials such as polysaccharides and proteins to obtain new biomaterials with favorable properties [10]. However, PVA films are too soft and contain a large number of hydroxyl groups in their structure, resulting in poor water resistance, which greatly limits their applications. There are some studies on superhydrophobic materials [11] and nanostructured materials which have extreme water repellency and self-cleaning properties [12]. In this study, polyvinyl alcohol and chitosan were modified by chemical crosslinking to improve the mechanical properties and water resistance of the corresponding film. Vanillin (Van) is a natural organic compound widely used as a flavoring and preservative in the food, fragrance, and cosmetic industries owing to its sweet and creamy flavor [13]. Importantly, vanillin is classified as “generally recognized as safe” (GRAS) in food by the US Food and Drug Administration (FDA) and has been found to have potential biological activities, including antioxidant, anti-inflammatory [14], and antibacterial effects on the growth of *E. coli* colonies. Vanillin contains a monoaldehyde and a phenolic hydroxyl group [15] and is widely used as a crosslinking agent in the preparation of biological materials. In this paper, vanillin-crosslinked PVA/CS active packaging composite (VPC) films were prepared by using CS and PVA as matrix materials and vanillin as a crosslinking agent.

## 2. Materials and Methods

### 2.1. Materials

CS and PVA were purchased from Sinopharm Group Chemical Reagent Co., Ltd. (Shanghai, China) and Shanghai Aladdin Biochemical Technology Co., Ltd. (Shanghai, China), respectively. Vanillin and anthocyanin were purchased from Shanghai Yien Chemical Technology Co., Ltd. (Shanghai, China) All reagents were used directly, without any purification.

### 2.2. Equipment

The following instruments were employed in the experiments: ME204E electronic balance [accuracy 0.0001 g, Mettler Toledo Instruments (Shanghai) Co., Ltd. (Shanghai, China)]; DF-101S heat collection constant-temperature heating magnetic stirrer [Bangxi Instrument Technology (Shanghai) Co., Ltd. (Shanghai, China)]; DHG-9140A electric blast drying box (Shanghai Yiheng Scientific Instrument Co., Ltd. (Shanghai, China)); Cary 50 ultraviolet–visible spectrometer (Varian Corporation, USA (Palo Alto, CA, USA)).

### 2.3. Preparation of VPC Films

The films were prepared according to Yu’s method [16], with some modifications. One gram of chitosan was dissolved in an aqueous acetic acid solution (1% *v*/*v*) and stirred at 50 °C for 1 h. Then, 3 g of PVA was dissolved in 100 mL of deionized water and continuously stirred at 95 °C for 1 h to obtain a PVA solution. After that, the PVA and CS solutions were evenly mixed in 1:1 mass ratio. Vanillin (0, 1, 3, and 5% *w*/*v*) was then added to the mixture solution and stirred at 70 °C for 30 min to form a vanillin/PVA/chitosan solution, and glycerol (3% *v*/*v*) was added as plasticizer. Finally, after cooling the mixed solution to 40 °C, an anthocyanin solution (10 mg of anthocyanin dissolved in 1 mL of deionized water) was added and stirred evenly to obtain the film-forming solution. After the solution was left to stand until all bubbles disappeared, 25 mL of the film-forming solution was poured into a 90 mm petri dish and baked at 40 °C until dry. According to the concentration of vanillin added, the obtained films are labeled VPC0, VPC1, VPC3, and VPC5.

### 2.4. Characterization of the Films

#### 2.4.1. Scanning Electron Microscopy (SEM)

The cross-sectional morphology of the thin films was observed using a scanning electron microscope at 1500× magnification. Before testing, the film was fractured in liquid nitrogen and sputter-coated with gold.

#### 2.4.2. X-Ray Diffraction (XRD)

XRD measurements were carried out using a Cu K_α_ radiation source, a tube pressure of 40 kV, and a tube flow of 40 mA. The XRD patterns were collected in the 2*θ* range of 5° to 90° at a scanning rate of 2°/min.

#### 2.4.3. Fourier Transform Infrared (FTIR) Spectroscopy

Infrared scanning was performed using an FTIR spectrometer in attenuated total reflection (ATR) mode. The samples were scanned 32 times at a spectral resolution of 4000–650 cm^−1^ with a scanning interval of 4 cm^−1^.

#### 2.4.4. Thermogravimetric Analysis (TGA)

The thermal stability of the films was measured by a thermogravimetric analyzer. A 4–5 mg amount of film was weighed and placed in a crucible. The temperature was set to rise from 30 to 700 °C at a heating rate of 25 °C/min under nitrogen gas protection, and the gas flow rate was 20 mL/min.

### 2.5. Performance Testing

#### 2.5.1. Water Content (MC)

The film was cut into 2 cm × 2 cm pieces, and its weight was recorded (*M*_0_). The *M*_0_ film sample was dried to a constant weight in an oven at 105 °C, and the final weight (*M*_1_) was recorded. The MC of the film was calculated according to the following formula:

(1) $$\mathrm{MC}(\%)=(M_{0}-M_{1})/M_{0}\times 100$$

#### 2.5.2. Water Vapor Transmittance (WVTR)

The WVTR of thin films was measured and calculated according to the methods reported by Kurabetta et al. [17]. The test was conducted at standard atmospheric pressure (75%RH). The film was cut into 30 × 30 mm^2^ sections, wrapped around the mouth of a glass vial (diameter 17 mm) containing 20 cm^3^ of deionized water, and sealed with Teflon tape. The initial weight (*W*_1_) of the glass vial was recorded and held for 24 h in a 40 °C electric blast drying oven (DHG-9140A). Then, the glass vial was removed from the oven, its final weight (*W*_2_) was recorded, and the WVTR of the active film was calculated according to the following formula:

(2) $$\mathrm{WVTR}\left(\%\right)=\frac{W_{1}-W_{2}}{A\times T}$$

where *T* is the drying duration (24 h), and *A* is the area of the mouth of the glass bottle.

#### 2.5.3. Light Transmittance

The transmittance and transparency of the films were determined by UV–vis spectroscopy. The film was cut into 1 cm × 2 cm sections, placed in a quartz cuvette, and the absorbance was measured at 600 nm. The light transmittance of the film was calculated by the following formula:

(3) $$T\%={\mathrm{Abs}}_{600}/x$$

where *Abs*_600_ is the absorbance value at 600 nm, and *x* is the thickness of the film (mm).

#### 2.5.4. Water Contact Angle (WCA)

The surface hydrophobicity/hydrophilicity of the active films was measured using a WCA analyzer (Chengde Dingsheng JY-82C video contact angle tester (Chengde, China)) at room temperature.

#### 2.5.5. Thickness and Mechanical Properties

The thickness of the active films was measured using a digital micrometer (Deli) with an accuracy of 0.001 mm. Six data points were read from different locations, and their average was taken as the film thickness. The mechanical properties of the films were measured by an electronic universal testing machine (American Instron 5982, (Boston, MA, USA)) according to the ASTM D 882-92 standard [18]. The film was cut into 25 mm × 100 mm sections, followed by stretching at a crosshead speed of 1 mm/min with an initial clip distance of 50 mm. Three parts of the same film sample were analyzed, and the results were expressed as the mean ± SD (*n* = 3) at room temperature. The mechanical properties of the films, including the tensile strength (TS) and elongation at break (EB), were calculated using a pre-installed software.

### 2.6. Antioxidant Properties

A DPPH (D) free radical scavenging test kit was used to determine the antioxidant effect of the films. A 100 μL volume of film-forming solution was added with 900 μL of extraction solution, swirled and stirred well by vortex oscillation, and centrifuged at 10,000 rpm for 10 min at room temperature; then, the supernatant was collected and placed on ice prior to the measurements. Then, 25 μL of supernatant and 975 μL of working liquid were mixed and allowed to stand for 30 min at room temperature, away from light, and the absorbance was measured at 515 nm. At the same time, the blank tube, measuring tube, control tube, and positive control tube were set, and each measuring tube was paired with a control tube. The DPPH free radical clearance rate of the film was calculated according to the following formula:

(4) $$D\%=\left[[A\;\mathrm{blank}-(A\;\mathrm{measure}-A\;\mathrm{control})]/A\;\mathrm{blank}\right]\times 100\%$$

where *A blank* is the absorbance value of blank tube, *A measure* is the absorbance value of measuring tube, and *A control* is the absorbance value of control tube.

### 2.7. pH Sensitivity

The film sample was soaked at room temperature in a pH 3.0–11.0 buffer solution for 5 min and then removed and photographed with a mobile phone (Apple 15pro) under the same light source with fixed parameters.

### 2.8. Statistical Analysis

The data were analyzed with the Excel 2016 software and mapped using the Origin 2021 program.

## 3. Results and Discussion

The aldehyde group in vanillin and the amino group in chitosan formed a network structure through a Schiff base reaction. The mechanism of aldehyde crosslinking was based on the formation of an imine bond, which is known as a Schiff base, between the aldehyde group of vanillin and the amino groups of the chitosan chains. Additionally, the hydroxyl groups of vanillin formed additional hydrogen bonds with the hydroxyl or amino groups of chitosan, promoting the cross-linking effect.

### 3.1. Characterization of Films

#### 3.1.1. SEM Analysis

**Figure 1:**
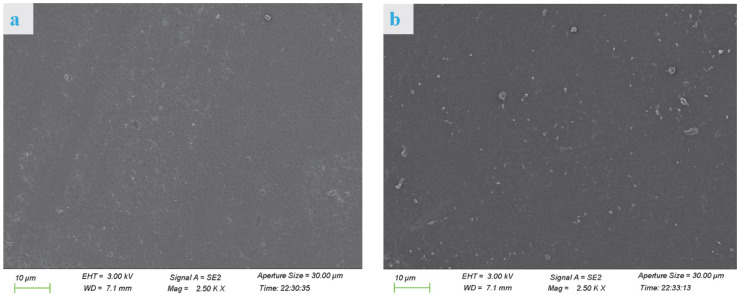

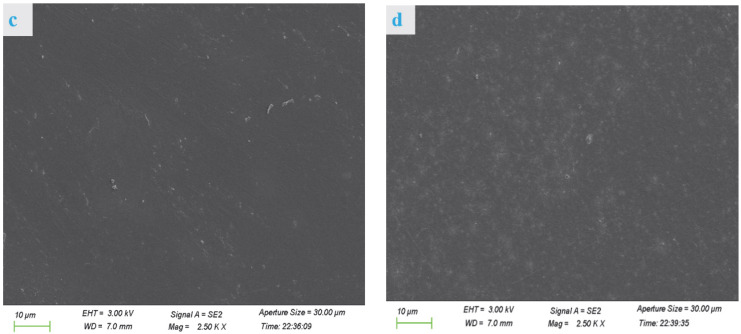
SEM micrographs of VPC0 (**a**), VPC1 (**b**), VPC3 (**c**), and VPC5 (**d**) films.

The cross section of the VPC0 film (Figure 1a) was relatively smooth with some visible protrusions and pores, while the VPC1 film (Figure 1b) showed no pores and increased protrusions, indicating that the crosslinking agent had a significant influence on the internal microstructure of the film. The cross sections of the VPC3 films (Figure 1c) had a significantly denser and smoother appearance than those of the other films, indicating that vanillin was successfully loaded into the film matrix and exhibited a certain degree of adhesion. The VPC5 film (Figure 1d) still had a compact appearance but presented a higher number of light spots. The differences in the cross sections of the films may be attributed to their different numbers of hydrogen bonds and Schiff base densities. All the films showed relatively continuous and dense cross-sectional structures, indicating the compatibility of vanillin with PVA/CS blends. This is consistent with the results of Yu et al. [16].

#### 3.1.2. XRD Analysis

**Figure 2:**
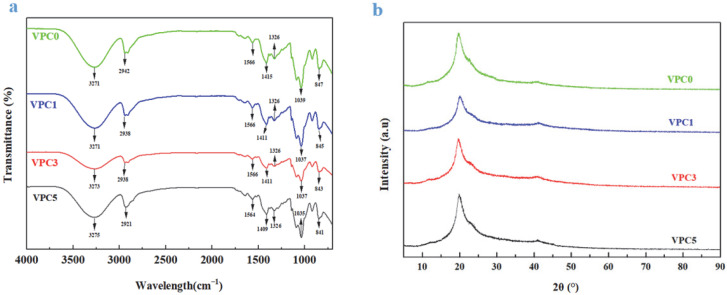
FTIR spectra (**a**) and XRD patterns (**b**) of VPC films.

The XRD patterns of the thin films are shown in Figure 2b. The four curves are similar. The main characteristic diffraction peaks of VPC0, VPC1, VPC3, and VPC5 were located at 2*θ* = 19.72°, 20.02°, 19.66°, and 19.84°, and the corresponding crystallinities were 31.48%, 49.45%, 38.37%, and 34.90%, respectively. Based on the above results, the active films with added vanillin had a higher crystallinity compared with VPC0; however, the crystallinity of the active films tended to decline with increasing vanillin concentration, which may be due to the enhanced crosslinking. The chemical bond between the hydroxyl group of PVA and the aldehyde group of Van results in a tighter network structure [19], which greatly limits the movement of PVA molecules, thus leading to a decreased crystallinity, which is also consistent with the findings of Yu et al. [16].

#### 3.1.3. FTIR Analysis

The interaction and functional group changes of Van-added PVA/CS films were characterized by FTIR spectroscopy. The FTIR spectra of the VPC thin films are shown in Figure 2a. The characteristic absorption peaks of all active films were similar, and no new absorption peaks were observed. It showed that vanillin was well integrated into the film and had good compatibility with other substances. Vanillin altered the senses of the active films, which gave off a slight vanillin smell. The peaks at wavenumbers between 3600 and 3100 cm^−1^ were attributed to the –OH stretching vibration, whereas those near 2900, 1566, 1326, and 1035 cm^−1^ corresponded to the –CH [20], –NH, –CN, and –CO [21] stretching vibrations, respectively. As shown in the figure, no new peaks appeared in the spectra of the four active films, indicating that no new chemical bonds were formed between the three components and confirming that the addition of Van did not change the structure of PVA and CS. Moreover, we found that the peak positions shifted with increasing Van concentration: the –OH stretching band moved to a higher wavenumber, and the peak value shifted to the left, whereas the bands corresponding to the –CH, –NH, –CN, and –CO stretching vibrations moved to lower wavenumbers, and the peak values shifted to the right; this may be due to the addition of Van, which increased the content of –OH. Strong hydrogen bonds were formed with the hydroxyl and amino groups on PVA and CS, resulting in an improved intermolecular compatibility. The formation of hydrogen bonds is expected to improve the mechanical properties of the film [22].

#### 3.1.4. Thermal Stability

**Figure 3:**
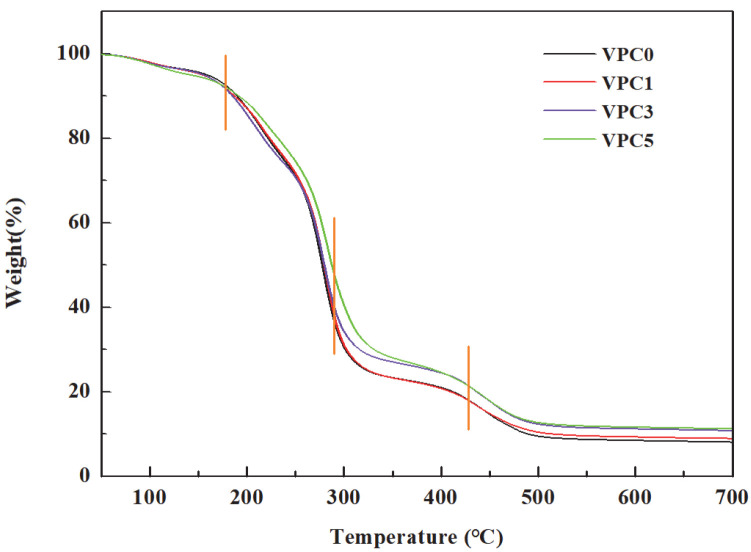
TGA curves of VPC films.

Thermogravimetric analysis was used to evaluate the thermal stability of the film. As shown in Figure 3, the four films showed similar stepwise weight loss patterns. The first weight loss stage of the film ranged from 100 to 170 °C, which may be due to the evaporation of surface-adsorbed water. In the second stage, from 170 to 280 °C, the four films showed a large range of weightlessness, which may be attributed to the loss of bound water, glycerin, and other low-molecular-weight compounds. The third stage, ranging from 280 to 430 °C, was the main film degradation stage, in which the PVA/CS structure was decomposed into small fragments through the occurrence of chain breaking reactions [23]. The fourth stage, between 430 and 500 °C, was related to the thermal degradation of hydroxyl groups and additives in the chitosan molecules. We found that the thermal stability of VPC5 was the lowest below 170 °C and remained the highest above 170 °C and below 400 °C. Above 250 °C, the thermal stability of VPC3 began to increase and matched that of VPC5 above 400 °C. Therefore, in the whole temperature range, VPC0 and VPC1 exhibited relatively poor thermal stabilities, whereas VPC3 and VPC5 showed different performances in different temperature ranges. The addition of Van enhanced the thermal stability of the film, possibly due to the generation of hydrogen bonds, as well as to Van reducing the water content of the film and interacting with the film substrate to improve its heat resistance.

### 3.2. Analysis of Physical Properties

#### 3.2.1. MC Analysis

**Figure 4:**
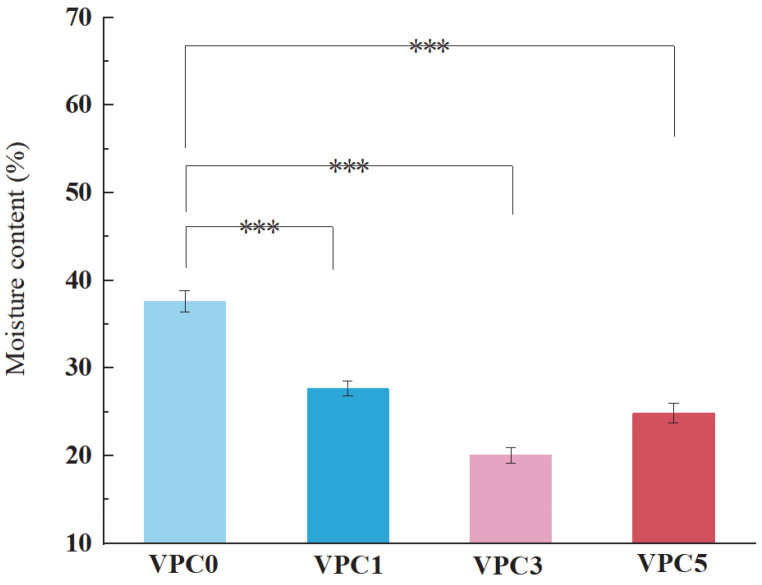
MC values of VPC films. *** *p* ≤ 0.001.

The moisture contents of the films are shown in Figure 4. Compared with the VPC0 film (37.53%), the moisture content of the other films decreased significantly after adding Van (27.68% for VPC1) and further increased after adding 5% *w*/*v* Van (24.87%). This is because the crosslinking mechanism is based on the formation of Schiff bases between the monoaldehyde and amino groups of the macromolecular chain, and, at the same time, the hydroxyl group of the crosslinking agent can form additional hydrogen bonds with the hydroxyl or amino group in the macromolecular chain, enhancing the crosslinking effect. The compact structure between the macromolecules does not allow the penetration of water molecules, thus reducing the moisture content of the film, which is consistent with the results of ref. [24]. However, when the crosslinking is too high, the uneven force exerted by the compact structure leads to different pore sizes, resulting in an increased water content of the film.

#### 3.2.2. WVTR Analysis

**Figure 5:**
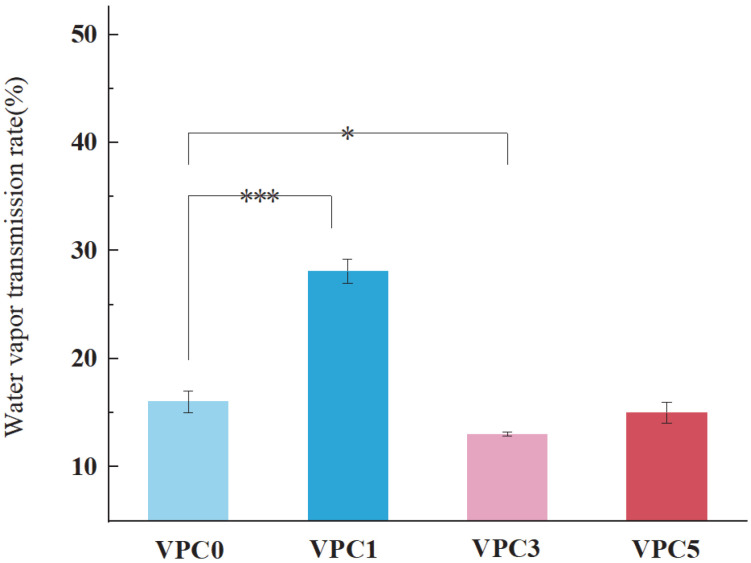
WVTR values of VPC films. * *p* ≤ 0.05, *** *p* ≤ 0.001.

The water vapor transmittance is an important index to measure the performance of active packaging films. Theoretically, a lower water vapor transmittance of an active packaging film indicates a better preservation effect [25]. This is because a lower WVTR of the active packaging film results in reduced water loss in the food product or the prevention of more water molecules from penetrating into it, achieving a better preservation effect [26]. As shown in Figure 5, compared with the vanillin-free VPC0, the WVTR of the VPC1 film with added vanillin increased by almost 12%, which may be related to the presence of a large number of hydrophilic phenolic hydroxyl groups in Van. With an increasing concentration of added Van, the WVTR value first decreased and then increased, suggesting that the degree of crosslinking altered the internal microstructural properties of the film, such as porosity and adhesion, preventing water vapor from passing through the food, thus suppressing water migration from the interior to the surface of the food product and achieving a barrier effect.

#### 3.2.3. Light Transmittance Analysis

**Figure 6:**
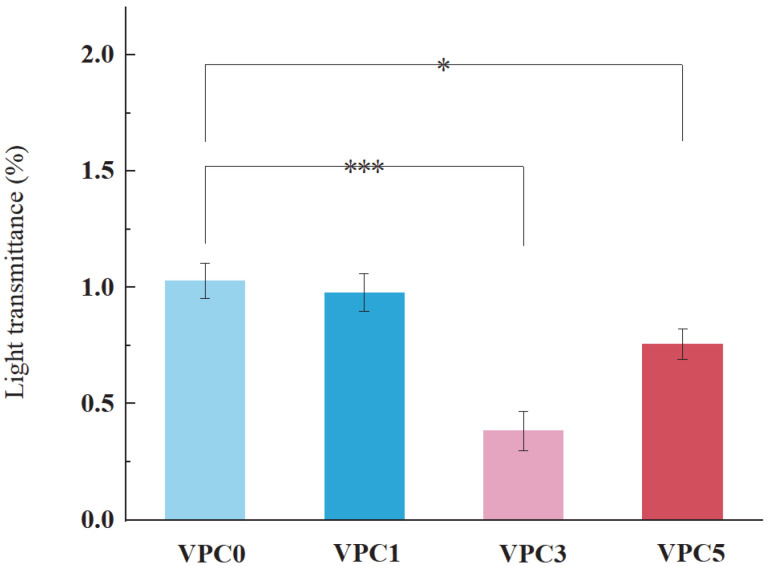
Light transmittance of VPC films at 600 nm. * *p* ≤ 0.05, *** *p* ≤ 0.001.

Ultraviolet radiation causes photochemical reactions in foods, resulting in color fading and retrogressed flavor and nutritional quality. Films which have low light transmittance can protect foods from photo-oxidation and extend the shelf life of food by maintaining quality [27]. The resistance to UV–visible light irradiation is an important property of active food packaging films [23], whose transmittance reflects their performance as a UV–visible light barrier. Figure 6 shows the UV–visible transmittance of the active films. The UV–visible light barrier performance of the active film increased with increasing vanillin content. In general, the transmittance of the four films ranged from 0.38% to 1.03%. According to the trend shown by these values, the addition of vanillin had a certain effect on the transmittance of the films, and the VPC3 film showed the best UV–visible light barrier performance.

#### 3.2.4. WCA Analysis

**Figure 7:**
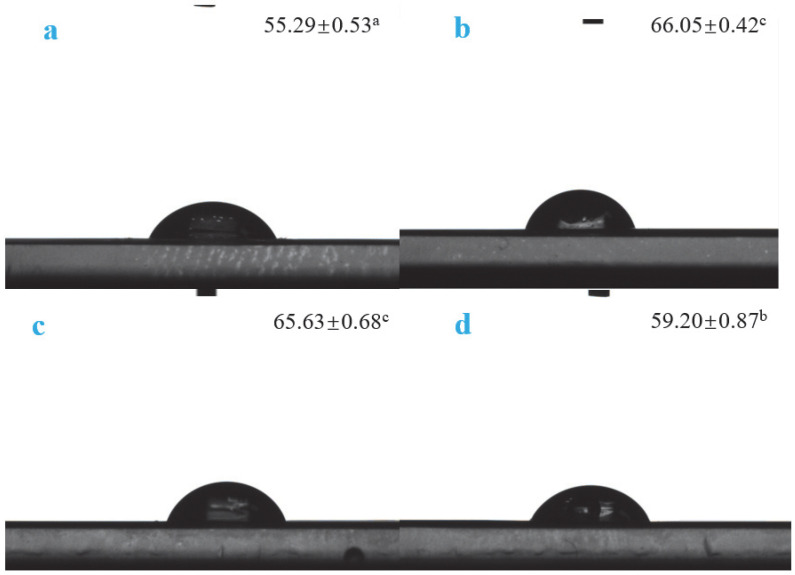
WCA values of VPC0 (**a**), VPC1 (**b**), VPC3 (**c**), and VPC5 (**d**) films. Different letters ‘^a^’, ‘^b^’, ‘^c^’ indicate the significant differences.

The water contact angle reflects the wettability of a film [28] and is an important index to characterize the water resistance of the film surface. In general, biopolymer membranes with water contact angles lower and higher than 60° are considered hydrophilic and hydrophobic, respectively [29]. The experimental data show that, as shown in Figure 7, after adding Van, the WCA values of the active films increased, and those of VPC1 and VPC3 were >60°, indicating a good hydrophobicity. Although the water contact angle of VPC5 was <60°, the measured value was significantly higher than that of VPC0, indicating that the addition of vanillin improved the hydrophobicity of the active film. This may be because chitosan contains a large number of hydroxyl/amino groups, which form hydrogen bonds with water molecules [30]. Upon the addition of vanillin, its phenolic hydroxyl group can form strong hydrogen bonds with the hydroxyl/amino group of chitosan, thus hindering the binding of water molecules with chitosan and improving the hydrophobicity of the film. As the active film has higher hydrophobicity, liquid water cannot wet the pore walls of the intermolecular spaces to form a continuous flow, which prevents its passage through the film.

#### 3.2.5. Analysis of Mechanical Properties

Thickness is an important factor when evaluating the physical properties of thin films. The thickness of the investigated films, shown in Table 1, ranged from 0.12 to 0.20 mm. The analysis of the data in the table shows that the thickness of the film first increased and then decreased after adding increasing concentrations of vanillin. Compared to the VPC0 film, the VPC1, VPC3, and VPC5 films had higher, similar, and lower thickness values, respectively. Although the thickness of the films changed, their values showed little difference. This difference may be attributed to the change in the overall fluidity due to the different concentration of the film-forming solution, the slight variation in the position of the film-forming container during the drying process, and the different thicknesses of the prepared films [31]. Studies have shown that the thickness of the film depends on the solid content in its composition and on the preparation conditions [32,33]. In this study, the CS and PVA components used in the film preparation were mixed in the same proportion, and the added concentration of Van was not significantly different; hence, the thickness of the film was not significantly affected by the Van concentration [23]. Compared with VPC0, the tensile strength of the vanillin-added active film was reduced, indicating that the hydroxyl group of vanillin interacted with the film matrix to form hydrogen bonds, resulting in a more cohesive structure that strengthened the film polymer matrix. At the same time, the elongation at break of the active film with added vanillin was significantly reduced, indicating that the addition of vanillin reduced the fluidity of the film matrix and limited the movement of macromolecules within it, which may be due to the uniform network structure formed by the vanillin-added active film. This is consistent with the results of Wang [13], who added vanillin to a PVA/κ-carrageenan crosslinked film, and Tao [34], who studied the addition of phloretin to a chitosan/PVA composite film.

### 3.3. Analysis of Antioxidant Properties

**Figure 8:**
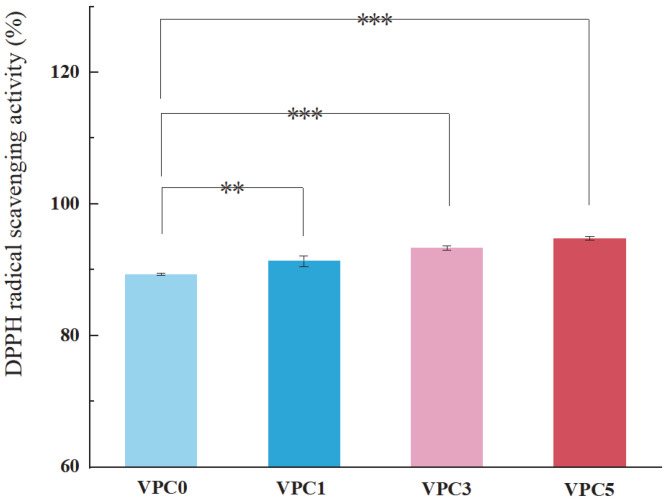
Antioxidant activity of VPC films measured by DPPH assay. ** *p* ≤ 0.01, *** *p* ≤ 0.001.

Antioxidant activity is another important functional property of active packaging films. A high antioxidant capacity of the film can slow down the oxidation reaction of food products, prevent their metamorphism and rancidity during storage and transportation, and maintain their nutritional composition and taste. If the films inhibited the rancidity of foods that would mean that the preservation effect was improved to a certain extent. The evaluation of the free radical scavenging ability is one of the standard methods for determining the antioxidant properties of thin films [35], with a higher free radical clearance rate denoting a better antioxidation performance of the film [36]. As shown in Figure 8, as the concentration of vanillin increased, the free radical scavenging ability of the film was enhanced, and the antioxidant capacity gradually increased; this indicates that vanillin had a positive effect on the antioxidant capacity of the film, which may be due to the large number of phenolic hydroxyl groups on the surface of vanillin that reduced or inhibited free radicals by transferring hydrogen atoms, thereby improving the corresponding scavenging abilities [36].

### 3.4. pH Sensitivity

**Figure 9:**
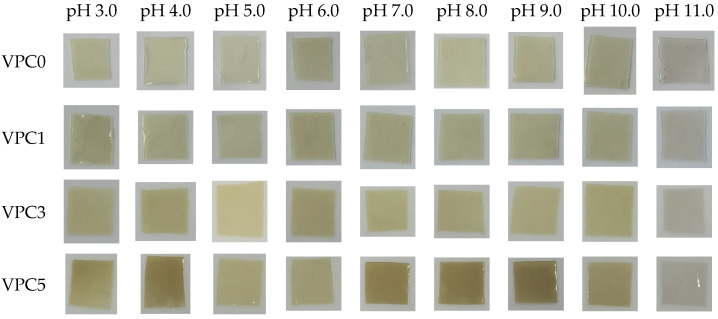
pH sensitivities of VPC films.

Changes in pH express changes in food quality in a sense, and the pH indicates the freshness of food [37]. Rancidity in most animal products results in a pH range of about 6.0–8.0 [38]. Figure 9 shows the color of the films in different pH buffer solutions. The results show that the four films had a good pH response. At pH 7.0, the color of the film was the closest to that observed in a neutral environment; hence, pH 7.0 was used as the reference group. At pH 7.0, the color of the VPC0 film was the lightest; then, the color of the VPC films became deeper with increasing vanillin concentration, with the VPC5 film showing the deepest color. As the acidity decreased (pH 3.0–6.0), the color of all films changed from dark to light. When the pH increased from 8.0 to 11.0, all films exhibited further color changes from dark to light. At pH 11.0, the four films displayed the lightest (almost transparent) color. The above results show that the four films had significant color responses in different pH buffer solutions, and exhibited strong pH sensitivity, with VPC5 displaying the most significant changes which were visually detected.

## 4. Conclusions

An active packaging composite film based on vanillin-crosslinked polyvinyl alcohol/chitosan was prepared in this study. The aldehyde group of Van reacted with the amino group of CS to form a Schiff base, and the phenolic hydroxyl group of Van formed a strong hydrogen bond with the amino group of CS. The multistage crosslinking reaction resulted in the film becoming denser and smoother. The antioxidant capacity of chitosan and vanillin can effectively extend the storage time of food products. Therefore, the present results show that the addition of vanillin can expand the functionalities of PVA/CS film materials, endowing vanillin-crosslinked PVA/chitosan active films with broader application prospects as packaging materials for food preservation.

## Figures and Tables

**Table 1:** Mechanical and physical properties of VPC films.

Sample	Thickness (mm)	Tensile Strength (MPa)	Elongation at Break (%)
VPC0	0.15 ± 0.021 ^a^	22.25 ± 2.27 ^a^	597.19 ± 75.02 ^a^
VPC1	0.20 ± 0.032 ^b^	17.98 ± 2.25 ^b^	453.34 ± 68.20 ^b^
VPC3	0.15 ± 0.012 ^a^	19.91 ± 2.99 ^c^	489.25 ± 82.04 ^c^
VPC5	0.12 ±0.031 ^c^	20.18 ± 4.23 ^d^	398.14 ± 118.70 ^d^

Results are presented as mean ± SD (*n* = 3). Different letters in the same column indicate the significant differences (*p* ≤ 0.05).

## Data Availability

The original contributions presented in this study are included in this article. Further inquiries can be directed to the corresponding author.
